# Bulk filling of Class II cavities with a dual-cure composite: Effect of curing mode and enamel etching on marginal adaptation

**DOI:** 10.4317/jced.51558

**Published:** 2014-12-01

**Authors:** Jose Bahillo, Tissiana Bortolotto, Miguel Roig, Ivo Krejci

**Affiliations:** 1 DDS, MsC, Master Esthetic Dentistry. Universitat Internacional de Catalunya, Department of Operative Dentistry and Endodontics, Sant Cugat del Valles, Barcelona, Spain; 2 Dr. Med. Dent., PhD, Senior Lecturer. Division of Cariology and Endodontology, School of Dentistry, University of Geneva, Geneva, Switzerland; 3 MD, DDS, PhD, Professor and Director. Department of Restorative Dentistry and Endodontics, School of Dentistry, Universitat Internacional de Catalunya, Sant Cugat del Valles, Barcelona, Spain; 4 Prof. Dr. Med. Dent., Professor and Chairman. Division of Cariology and Endodontology, President, School of Dentistry, University of Geneva, Geneva, Switzerland

## Abstract

Objectives: This study attempted to find a simple adhesive restorative technique for class I and II cavities on posterior teeth. Study Design: The tested materials were a self-etching adhesive (Parabond, Coltène/Whaledent) and a dual-cure composite (Paracore, Coltène/Whaledent) used in bulk to restore the cavities. Class II MO cavities were performed and assigned to 4 groups depending on the orthophosphoric acid (H3PO4) conditioning of enamel and polymerization method used (chemical or dual). Specimens were subjected to quantitative marginal analysis before and after thermo-mechanical loading. Results: Higher percentages of marginal adaptation at the total margin length, both before and after thermo-mechanical loading, were found in groups in which enamel was etched with phosphoric acid, without significant differences between the chemically and dual-cured modes. The restorations performance was similar on enamel and dentin, obtaining low results of adaptation on occlusal enamel in the groups without enamel etching, the lowest scores were on cervical dentin in the group with no ortophosphoric acid and self-cured. Conclusions: A dual-cure composite applied in bulk on acid etched enamel obtained acceptable marginal adaptation results, and may be an alternative technique for the restoration of class II cavities.

** Key words:**Dual-cure composite, bulk technique, class II restoration, selective enamel etching, marginal adaptation.

## Introduction

Amalgam has been the material of choice worldwide for class I and class II restorations for more than a century due to its high strength, good wear resistance, low technique sensitivity and low cost (1). However, the lack of esthetics, corrosion and difficult bonding to tooth structure requiring the removal of sound structure to gain on macromechanical retention resulted in the need to find an amalgam substitute for the esthetic restoration of decayed teeth.

Advances in adhesive technology made during the last 40 years have simplified dental procedures and given a more esthetic result to patients and a conservative alternative treatment to clinicians (2). While the use of amalgam for the restoration of posterior cavities is declining, composite resins are being more often used with almost no differences in terms of clinical longevity (3).

Previous studies of composite class II resin restorations have shown that their clinical longevity is adversely affected by several factors such as achieving a good proximal contact (1), lack of cervical enamel (4), difficulties for beveling proximal enamel margins, a limited wall flexibility to allow for a certain compensation of polymerization contraction (5), and poor composite adaptation at the gingival portion if margins are subgingivally located (6).

Polymerization contraction of dimethacrylate-based composites ranges from 2-6% of volumetric shrinkage (7). Various clinical methods have been proposed to reduce shrinkage stress. These methods include the regulation of curing light intensity (8), a flowable resin liner application (9), the elaboration of an indirect resin restoration (10), and the use of a sophisticated incremental layering technique (1). Another alternative has been the use of an open-sandwich technique restoration (11,12). In this procedure, a resin-modified glass-ionomer cement [RMGIC] or a glass ionomer is placed as a base covering the entire proximal box, to complete the restoration to full anatomic form and function, and then a top layer of a light-cured composite resin is placed. However, despite relatively good short-term clinical results, a noticeable dissolution of RMGIC has been reported after six years (12).

Dual-cured resin composites have been mainly used as a core material for the reconstruction of non-vital teeth (13), and as dentin substitute in the open sandwich filling technique (11,14). Some advantages of using dual-cured composites as filling material would be the possibility of a bulk insertion, clinical time saving, the achievement of polymerization in deep areas due to chemical curing and the development of lower contraction stresses (15).

A previous study reported that the tooth-restoration complex is more prone to fail at the interface rather than in the composite or tooth material (16). Therefore, assessing the integrity of the margins before and after a thermo-mechanical fatigue test would be discriminative enough to show differences among the different restorative techniques.

In an attempt to find a restorative material that could potentially be used as an amalgam substitute, i.e. an adhesive material that can be applied in bulk with a simple restorative technique, we evaluated the marginal integrity of cavities entirely restored with a dual-cured resin composite. The null hypotheses tested were that [i] The differences in the etching procedure would not affect the marginal adaptation before and after thermo-mechanical loading and [ii] Marginal adaptation would not be affected by the polymerization mode [chemical or dual-cure].

## Material and Methods

The materials used for the study consisted of a chemically-cure self-etch adhesive system [Parabond, Coltène/Whaledent, Altstätten, Switzerland] composed by a non-rinse conditioner and 2 liquids [A and B] that need to be mixed before application, and a dual-cure radiopaque core material [Paracore, Coltène/Whaledent] ( Table 1). Thirty-two sound extracted human molars with complete apexification were selected for the study. After scaling and pumicing, the teeth were mounted on custom-made specimen holders with their roots in the centre using a cold-polymerizing resin [Technovit 4071, Heraeus Kulzer GmbH, Wehrheim, Germany]. Prior to the mounting procedure the apices were sealed with two coats of nail varnish. To simulate dentinal fluid flow, a cylindrical hole was drilled into the pulpal chamber approximately in the middle third of the root and a metal tube with a diameter of 1.4 mm was then adhesively luted using a dentinal adhesive [Syntac Classic, IvoclarVivadent AG, Schaan, Liechtenstein]. The pulpal tissue was not removed. This tube was connected by a flexible silicone hose to an infusion bottle placed 34 cm vertically above the test tooth. The infusion bottle was filled with horse serum [PAA Laboratories GmbH, Linz, Austria] and phosphate-buffered saline solution [PBS; Oxoid Ltd, Basingstoke, Hampshire, England] diluted in a 1:3 ratio under a hydrostatic pressure of about 25 mm Hg. Twenty-four hours before starting the cavity preparations, by using a three-way valve, the pulp chambers were evacuated with a vacuum pump and subsequently bubble-free filled with the above solution. As of this moment, the intrapulpal pressure was maintained at 25 mm Hg throughout the testing, i.e. during cavity preparation, restoration placement, finishing and stressing (17,18).

**Table 1:**
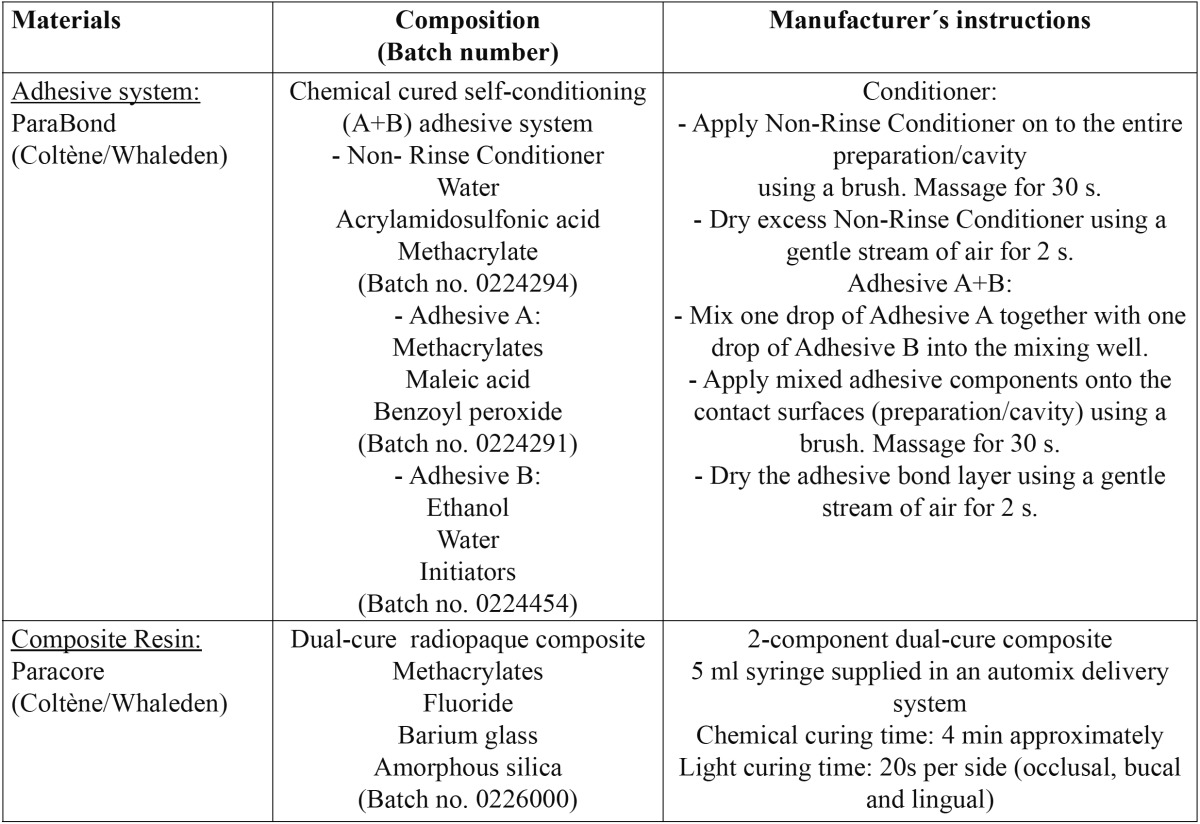
Materials, composition and manufacturer´s instructions.

Standardized box-shaped mesio-occlusal class II cavities with cervical margins located on dentin were prepared on each tooth using diamond burs with a grain size of 80 microns [Diatech Dental, Coltène/Whaledent] and finished with diamond burs with a grain size of 40 microns [Diatech Dental] under 12x magnification. The dimensions of the cavities were 4 mm height, 4 mm of bucco-lingual width, 2.5 mm mesio-distal depth and approximately 1.5 mm below the cemento-enamel junction.

In groups 1 and 2 the adhesive system was applied following manufacturer recommendations [that is, without previous acid etching of enamel] ( Table 1). In groups 3 and 4 enamel was etched with 37.5% H3PO4 [Kerr, Scafati, Italy] for 30 s, rinsed and air-dried. The non-rinse conditioner was then applied on dentin [enamel was avoided because it had been previously etched with phosphoric acid]. After the application of the mixed A and B adhesive components, cavities were filled with the build-up composite in one single layer. In groups 1 and 3 the restorations were left undisturbed for at least 4 min to enable the materials self-cure. In groups 2 and 4 they were immediately light cured with a curing device [L.E.D. Demetron II, Serial No: 792026758, Kerr, Orange, CA, USA] with a relative intensity of 800 mW/cm2 [Curing Radiometer, Demetron Research, Danbury, CT, USA] 20 sec per side [occlusal, buccal and lingual]. The same operator performed the restoration of all groups.

**Figure 1:**
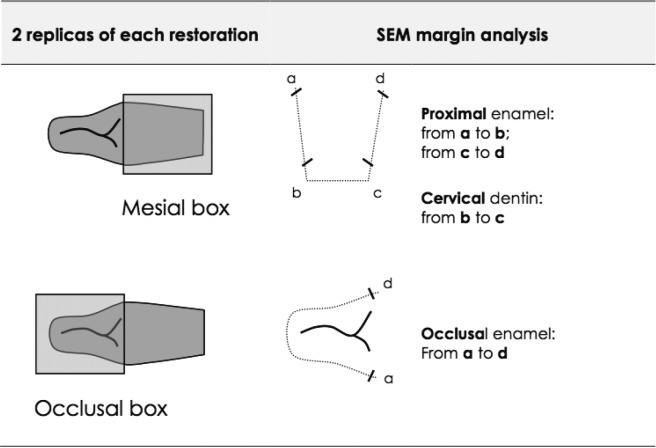
Schematical representation of the quantitative evaluation for the SEM marginal analysis, assessing the % of continuous margins at the occlusal enamel proximal enamel and cervical dentin margins. The average value of enamel and dentin marginal adaptation was calculated and reported as the total marginal length (TML).

One day after polymerization, finishing and polishing of the restorations was carried out using flexible discs [SofLex PopOn, 3M ESPE AG, Seefeld, Germany] for proximal and fine-grain diamond burs for the occlusal surface. Then, impressions with a polyvinylsiloxane material [President light body, Coltène/Whaledent] were made of each restoration and poured with epoxy resin [Epofix Resin, Struers, Willich, Germany] and 24 h after resin replicas were gold sputtered. They were subjected to a computer assisted quantitative margin analysis in a scanning electron microscope [XL20, Philips, Eindhoven, Netherlands] by using a custom made module programmed with an image processing software [Scion Image, Scion Corp, Frederik, USA]. For the quantitative evaluation, a blinded and trained lab technician examined the specimens (Fig. 1). The marginal quality was expressed in percentages of continuous margins [% CM] and reported for occlusal enamel margins, proximal enamel, cervical dentin and for the total marginal length [average value of enamel and dentin marginal adaptation], at each interval before and after loading. After storage for 7 days at 37°C in the dark, the teeth were loaded with simultaneous repeated thermal [600x from 5° to 55°C with a dwell time of 2 min] and mechanical stresses [240,000 chewing cycles at 1.7 Hz] by an antagonistic natural molar cusp with a maximum load of 49 Newtons under the constant simulation of dentinal fluid flow (19). After thermo-mechanical loading, the teeth were cleaned with toothpaste, rinsed with tap water and impressions were taken again in order to perform the marginal replicas for SEM analysis after loading (19).

## Statistical Analysis

Analysis of data was performed by using an analysis of variance test (ANOVA) of variance [ANOVA] for each variable separately for two time points [before and after loading] in SPSS 19 for Windows. Significant differences were observed between groups with Tukey HSD post-hoc pairwise comparasion. In all analyses, the level of significance was set at 0.05.

## Results

The scores of marginal adaptation expressed as percentages of continuous margins [% CM] attained by the different groups at the total margin length, occlusal, proximal and cervical margins are shown in  Table 2 [before load] and  Table 3 [after load].

**Table 2:**
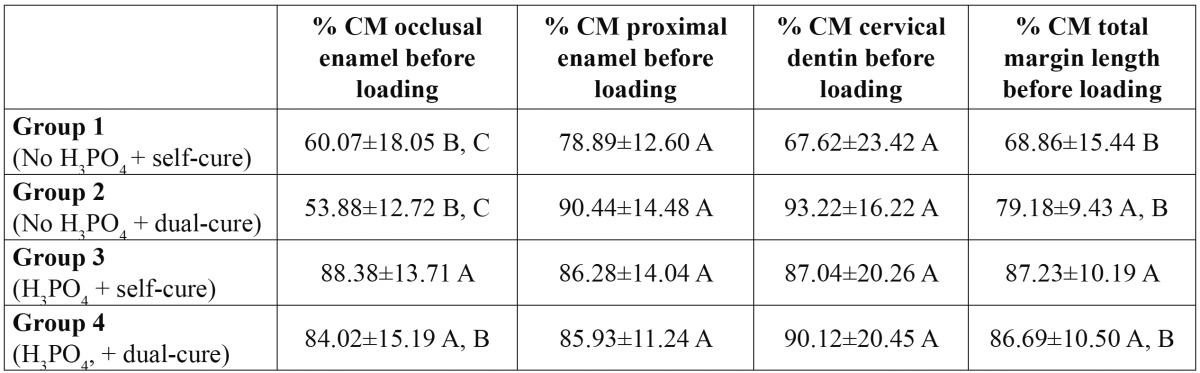
Mean ± Standard Deviation scores of each group before thermo-mechanical loading on occlusal margins, on proximal margins, on cervical margins and at the total margin length (average of occlusal, proximal and cervical marginal adaptation). Abbreviations. % CM: percentages of continuous margins. Levels connected by the same letter are statistically similar and apply to each column.

**Table 3:**
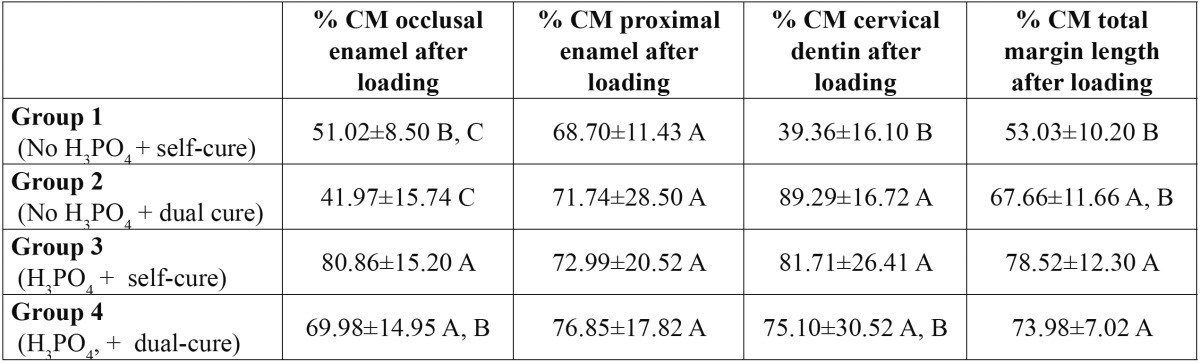
Mean ± Standard Deviation scores of each group after thermo-mechanical loading on occlusal margins, on proximal margins, on cervical margins and at the total margin length (average of occlusal, proximal and cervical marginal adaptation). Abbreviations. % CM: percentages of continuous margins. Levels connected by the same letter are statistically similar and apply to each column.

- Quantitative margin analysis

Before loading ( Table 2), higher % of CM were observed at the total margin length [TML] in the groups in which enamel margins were etched with phosphoric acid. At the different cavity segments [occlusal, proximal and cervical], lower % of CM were obtained at the occlusal margins in groups 1 and 2 where enamel was not etched with phosphoric acid, being statistically significant between group 2 with group 3 [*p*=0.001] and group 4 [*p*=0.004]. However, no significant differences between groups were seen at the proximal margins or cervical portion.

After loading, the highest scores of marginal adaptation at TML were attained, once again, by the groups that used H3PO4 ( Table 3). Statistically significant differences were obtained between group 1 and group 3 [*p*=0.002] and with group 4 [*p*=0.013]. At the cavity segments, we obtained again lower % of CM at the occlusal side in groups in which phosphoric acid on enamel was not used. No differences between groups were observed at the proximal enamel margins. However, at the cervical portion, the lowest % of CM were obtained in group 1 being statistically different from group 2 [*p*=0.008] and group 3 [*p*=0.024].

**Figure 2:**
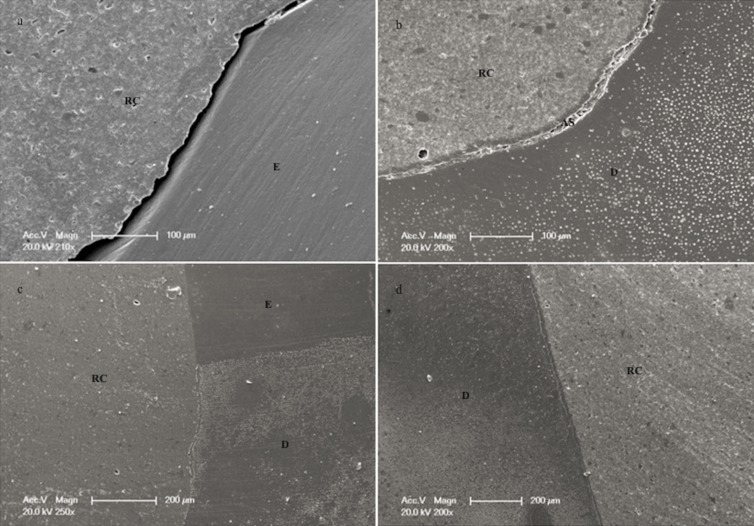
Representative SEM micrographs for the 4 groups evidencing continuous and non-continuous margins; a) SEM image of group 1 (No H3PO4 + self-cure) showing a clear open margin between enamel (E) and composite material (RC), a no orthophosphoric acid etching enamel surface may be related with the lower % of marginal adaptation at the occlusal portion in this group; b) SEM image of group 2 (No H3PO4 + dual-cure) at the cervical bottom part of the cavity, despite the good results obtained in this group at this segment, a non-continuous margin can be observed between dentin (D), adhesive system (AS) and composite (RC); c) SEM image of group 3 (H3PO4 + self-cure) at the CEJ level with an intact continuous margin, no interruption of the adhesive interface between resin composite (RC) with enamel (E) and dentin (D) could be observed, representing the high percentage of continuous margins; d) SEM Image of group 4 (H3PO4 + dual-cure) evidencing a close margin between dentin (D) and resin composite (RC), showing no differences on marginal adaptation in comparison with the self-cured approach.

Representative SEM images (Fig. 2) with margins on enamel and dentin after thermo-mechanical loading for the 4 groups [a, b, c, d]. In figure 2 section a, an open margin at the occlusal segment could be observed; this finding could be related to the fact that enamel was not etched with orthophosphoric acid. In group 2, a non-continuous dentin margin was detected at the cervical portion as shown in figure 2 section b. Continuous and intact margins could be seen in figure 2 section c for the group 3 at the CEJ, representing the high percentages of adhesion. Dentin continuous margins are shown in figure 2 section d, showing no differences between the self and dual-cured approach.

## Discussion

The rationale for including the evaluation of marginal adaptation before and after thermo-mechanical loading relies on the fact that both methodologies constitute and adequate model to assess the performance of adhesive interfaces (19-21). In respect to the materials, a chemically-cured two-step self-etching adhesive system was used with a dual-cured build-up composite. To simplify the adhesive procedure we could have selected a 1-bottle or simplified self-etching adhesive system. However, the literature reported incompatibility problems due to the chemical interaction between simplified-step adhesives and chemical or dual-cured composites (22,23). This incompatibility is attributed to an adverse chemical interaction between the acidic monomer and the tertiary amines present within the composite. These features were absent when two-step self-etch adhesive are used (24), justifying why in the present study simplified adhesive systems were avoided.

It is known from previous studies that when etch and rinse adhesives are used on enamel, a reliable and favorable bonding interface is produced (25). However, when using dual-cured composites in conjunction with dual-cured dental adhesives, the self-etching approach has been proved to perform better on dentin (26). Therefore, a selective enamel etching would ensure a more adequate bonding effectiveness even when a self-etching system is selected (27). The results of the present study confirmed the precedent statement as we obtained the highest % of CM at the total margin length, both before and after loading, when enamel margins where etched with phosphoric acid, independently of the polymerization method used, leading to reject the first null hypothesis.

Marginal adaptation at the occlusal enamel margins were lower for the groups 1 and 2 where the self-etching primer was applied on enamel without previous conditioning with phosphoric acid. In addition, unsupported enamel prisms [margins were not beveled] may have resulted in enamel fractures at the marginal level especially after loading (10).

Gap formation or microleakage at the tooth-composite interface is related with polymerization shrinkage and shrinkage stresses. This issue may induce postoperative sensitivity and secondary caries, leading to restoration failure (28). Minimizing those determinant factors would improve marginal cavity adaptation (29). Light intensity was the highest at the restorations’ surface decreasing the pre-gel phase and leading to contraction forces and materials’ shrinkage (8). This could explain the low results on occlusal enamel despite better scores of marginal adaptation on the proximal area.

Shrinkage strain rates of both self and light-curing modes would become almost identical after about 60 min for dual-cured core materials (8). In the present study, similar shrinkage stresses could have been developed on the self and dual-cured material at the late stages of polymerization, explaining why the percentages of continuous margins were similar in both groups, having to accepted the second null hypothesis.

Despite the similar marginal adaptation scores, when dual-cure composites are used, higher dentin bond strength are reported when light-cured as compared to self-curing (30). This could explain the lower results of marginal adaptation obtained at the cervical dentin in group 1, the self cured material would attain a lower degree of conversion and therefore, lower mechanical properties that would be evidenced at the marginal level in respect to the light cured ones.

In addition, final light polymerization would enhance significant mechanical properties, making the selection of a dual-cured composite an improvement over a self-cured or a light-cured at the gingival margin (11).

To conclude, materials insertion in one single layer, with the additional benefit of chemical cure in deep areas where light is attenuated has several clinical advantages in terms of handling, curing efficiency and time saving. Possibly, the restorative technique used in the present study could be a cost-effective alternative to traditional sophisticated adhesive restorative techniques, and a potential substitute to amalgam (31). Nevertheless, our methodology did not evaluate the wear resistance of such materials, which might be of clinical importance in intraoral conditions. In this perspective, future research should be also undertaken to assess if other dual-cured materials than the ones used in the present study experience a similar behavior in terms of marginal adaptation.

Within the limitations of the present study, the use of a dual-cure composite for the restoration of class II cavities, applied in bulk on phosphoric acid etched enamel, has provided the most reliable marginal adaptation.
